# Generation of *Plasmodium falciparum* parasite-inhibitory antibodies by immunization with recombinantly-expressed CyRPA

**DOI:** 10.1186/s12936-016-1213-x

**Published:** 2016-03-15

**Authors:** Paola Favuzza, Simon Blaser, Anita M. Dreyer, Guy Riccio, Marco Tamborrini, Ralf Thoma, Hugues Matile, Gerd Pluschke

**Affiliations:** Medical Parasitology and Infection Biology Department, Swiss Tropical and Public Health Institute, Basel, Switzerland; University of Basel, Basel, Switzerland; Roche Pharmaceutical Research & Early Development, Small Molecule Research, Roche Innovation Center Basel, F. Hoffmann-La Roche Ltd., Basel, Switzerland

**Keywords:** Malaria, CyRPA, Erythrocyte invasion, Blood-stage vaccines

## Abstract

**Background:**

The pathogenesis of malaria is primarily associated with blood-stage infection and there is strong evidence that antibodies specific for parasite blood-stage antigens can control parasitaemia. This provides a strong rationale for incorporation of asexual blood-stage antigen components into an effective multivalent malaria subunit vaccine. On the basis of available genome-wide transcriptomic and proteomic data, previously uncharacterized *Plasmodium falciparum* open reading frames were screened for new blood stage vaccine candidates. This has led to the identification of the cysteine-rich protective antigen (PfCyRPA), which forms together with PfRH5 and PfRipr a multiprotein complex that is crucial for erythrocyte invasion.

**Methods:**

Glycosylated and non-glycosylated variants of recombinant PfCyRPA were expressed and produced as secreted protein in mammalian cells. Adjuvanted formulations of purified PfCyRPA were tested to assess whether they can effectively elicit parasite inhibitory antibodies, and to investigate whether or not the glycosylation status affects antibody binding. For this purpose, two sets of PfCyRPA-specific mouse monoclonal antibodies (mAbs) have been raised and evaluated for functional activity.

**Results:**

Generated PfCyRPA-specific mAbs, irrespective of the immunogen’s glycosylation status, showed substantial parasite in vitro growth-inhibitory activity due to inhibition of erythrocyte invasion by merozoites. Furthermore, passive immunization experiments in *P. falciparum* infected NOD-*scid IL2Rγ*^*null*^ mice engrafted with human erythrocytes demonstrated potent in vivo growth-inhibitory activity of generated mAbs.

**Conclusions:**

Recombinantly expressed PfCyRPA tested as adjuvanted vaccine formulations in mice elicited antibodies that significantly inhibit *P. falciparum* asexual blood stage parasite growth both in vitro and in vivo. These findings render PfCyRPA a promising blood-stage candidate antigen for inclusion into a multicomponent malaria subunit vaccine.

**Electronic supplementary material:**

The online version of this article (doi:10.1186/s12936-016-1213-x) contains supplementary material, which is available to authorized users.

## Background

The World malaria report 2015 reported the reduction of malaria mortality rates by an impressive 48 % between 2000 and 2015 as a result of a major scale-up of vector control interventions, diagnostic testing, and treatment with artemisinin‐based combination therapy [1]. Despite these tremendous achievements, an estimated 214 million cases of malaria occurred globally in 2015, and the disease led to 438,000 deaths, mostly those of children under 5 years of age in Africa [1]. Limited efficacy achieved by subunit vaccine candidates, emerging anti-malarial drug resistances, along with reported insecticide resistances, underline the need of new tools to control and prevent malaria [2, 3]. In this perspective, the development of an effective malaria vaccine is recognized as one of the most promising approaches to conquer the disease. Despite decades of research, an effective vaccine against malaria has remained elusive. Anti-malarial vaccines can break the parasite life cycle at different stages: infection-blocking vaccines targeting hepatic stages, anti-morbidity vaccines targeting the asexual blood stages, and transmission-blocking vaccines targeting the sexual stages. To achieve effective protection, the ideal malaria vaccine is thought to target several steps of the parasite life-cycle in a multistage combination vaccine [4].

Clinical and experimental data support the feasibility of developing an effective malaria vaccine. Adults living in malaria endemic areas rarely experience malaria episodes: partial protection of adults is mediated by naturally acquired immunity, and protects against symptomatic disease and high‐density parasitaemia, but is not effective in offering sterile immunity [5]. Also, passive transfer of γ-globulin from semi-immune adults to malaria patients conferred a significant reduction of parasitaemia and recovery from clinical symptoms [6]. Those studies showed that immunity can be naturally acquired with exposure and indicated antibodies as crucial components of the protective immune response against asexual blood stage parasites. In this perspective, a multi-stage malaria vaccine should contain as one component antigen(s) that elicit antibody responses upon parasite presentation, leading to clearance of asexual blood stage parasites, and thus reducing the clinical symptoms.

Currently, with a total 25 projects in the pipeline [7], three candidate vaccines are in phase 2B clinical trials and one, the pre-erythrocytic subunit vaccine RTS,S/AS01, has completed phase 3 [8]. In infants aged 6–12 weeks at first vaccination with four doses of RTS,S reduced the number of cases of clinical malaria by 26 % to the end of the study over an follow-up of 38 months. Blood-stage vaccines, designed to elicit anti-invasion and anti-disease responses [9], are traditionally mainly based on a few protein candidate antigens: apical membrane antigen 1 (AMA1) [10–12], erythrocyte-binding antigen-175 (EBA-175) [13], glutamate-rich protein (GLURP) [14, 15], merozoite surface protein (MSP) 1 [16], MSP2 [17, 18] and MSP3 [19, 20] and serine repeat antigen 5 (SERA5) [21, 22]. These immunodominant antigens, highly expressed on merozoites surface or within apical organelles, are involved in the invasion process. Unfortunately, AMA1 and MSP1, the most advanced blood-stage vaccines, have not demonstrated effective protection in African children [10, 16, 23], probably due to their highly polymorphic nature [24]. Genetic variability, extensive polymorphism and antigenic complexity in immunodominant antigens represent major obstacles in the development of an effective blood-stage malaria vaccine [25–27]. Identifying and designing antigens able to induce strain-transcending immune responses, which cover antigenic diversity remains a critical issue to be addressed. Since the *Plasmodium falciparum* genome was sequenced and annotated in 2002 [28], reverse vaccinology represents the most attractive strategy to rationally identify novel malaria vaccine candidates [29, 30]. On the basis of the large-scale genomic, transcriptomic, proteomic and comparative data from *Plasmodium* spp. that have become available, new antigens with great potential as blood-stage vaccine candidates have been discovered [31].

Among the newly characterized proteins, the cysteine rich protective antigen (PfCyRPA) exhibited remarkable properties: PfCyRPA (1) elicits Abs that inhibit parasite growth in vitro and in vivo [32], (2) is highly conserved among *P. falciparum* isolates [32], (3) has limited natural immunogenicity, and (4) forms together with the reticulocyte-binding homolog 5 (PfRH5) and the PfRH5-interacting protein (PfRipr) a multiprotein complex crucial for *P. falciparum* erythrocyte invasion [33]. PfRH5 is currently regarded another leading blood-stage malaria vaccine candidate: it has been shown to induce invasion-inhibitory antibodies that are effective across common PfRH5 genetic variants and PfRH5-based vaccines can protect *Aotus* monkeys against virulent vaccine-heterologous *P. falciparum* challenges [34–37]. The PfCyRPA encoding gene *PFD1130w* is located in the subtelomeric region of chromosome 4 in close proximity to other genes playing a crucial role in the erythrocytes invasion, including *PFD1145c* that encodes for PfRH5 [36]. PfCyRPA is a 362-aa-long protein with a predicted molecular mass of 42.8 kDa, an N-terminal signal peptide, a C-terminal GPI-anchor motif and twelve cysteine residues, potentially involved in the formation of six disulfide bridges. PfCyRPA was identified as a promising blood-stage malaria vaccine candidate exploiting a cell-based approach that utilizes antigens expressed on the surface of mammalian cells for mouse immunization [38]. Since antigen-loaded cells are not suitable for human immunization, the study investigated whether invasion inhibitory anti-PfCyRPA antibodies could be raised by active immunization with purified recombinant PfCyRPA protein. In the present study, PfCyRPA was recombinantly-expressed in mammalian cells and adjuvanted vaccine formulations of purified PfCyRPA were tested for their potential to elicit antibodies that inhibit *P. falciparum* parasite growth in vitro and in vivo.

## Methods

### Bacterial strains and media

*Escherichia coli* strain Top10 (Life Technologies) was used for the amplification of plasmids. Bacteria were grown in LB medium containing 100 μg/ml ampicillin at 37 °C.

### Construction of expression plasmids

The expression vector which allows for the secretion of the recombinant PfCyRPA protein (aa 22–362) was generated by PCR-based mutagenesis [39–42] using the BVM_PFD1130W_FLAG_GP_His plasmid as template [38]. Briefly, a PCR product encompassing the bee-venom melittin secretion signal (BVM) and PfCyRPA aa 26–352 codon-optimized sequence, was amplified using GeneAmp^®^ High Fidelity PCR System (Life Technologies) and primer 4325 (5′-CAACTCCGCCCCATTGACGCA-3′) and 4326 (5′-GGTGTGGATGTTGTAAATGCCCTGGGA-3′). The hexa-his tag was amplified with primers 4329 (5′-GAGGAATTCCATCACCATCACCATCACTGATAA-3′) and 4330 (5′-AGGGCGATGGCCCACTACGT-3′). A double-stranded oligonucleotide encoding for PfCyRPA aa 353–362 was generated by oligos-annealing employing the complementary oligonucleotides 4327 (5′-ATTTACAACATCCACACCATCTACTACGCCAACTACGAGGAATTCCATCACCAT-3′) and 4328 (5′-ATGGTGATGGAATTCCTCGTAGTTGGCGTAGTAGATGGTGTGGATGTTGTAAAT-3′). In a second step, a ligation PCR was performed with the outermost primer pair (4325 and 4330) using a mixture of the three previously generated PCR amplicons. Eventually, the recombined PCR product was recloned by NheI and XhoI (New England Biolabs) resulting in plasmid pcDNA3.1_BVM_CyRPA(26–362)_6xHis. This expression vector allows the expression of PfCyRPA with a hexa-His tag as secreted protein via the BVM signal peptide (designated G-CyRPA). It contains the secretion signal of bee-venom melittin, the coding sequence of the protein of interest and a hexa-His tag.

The expression vector coding for the non-glycosylated PfCyRPA (N-CyRPA) was generated by site-directed mutagenesis (GenScript) resulting in the expression plasmid pcDNA3.1_BMV_CyRPA(26–362/N145Q-N322Q-N338Q)_6xHis.

### Culture of eukaryotic cells

FreeStyle 293-F cells (Thermo Fisher), a variant of human embryonic kidney cell line HEK cells, were cultured in suspension in serum-free medium (FreeStyle™ 293 Expression Medium, Thermo Fisher) at 37 °C in a humidified incubator with 5 % CO_2_. Shake flask cultures were run in 1 l shake flasks (Corning, 120 rpm, 5 cm diameter) and 10 l cultures were performed in fully instrumented Wave bioreactors (Sartorius, Melsungen) under controlled conditions (30 rpm, pH 7.2, 30 % DO).

### Recombinant protein expression and purification

FreeStyle 293-F cells were transfected with pcDNA3.1_BVM_CyRPA(26–362)_6xHis and pcDNA3.1_BMV_CyRPA(26–362/N145Q-N322Q-N338Q)_6xHis plasmids using a riDOM-based transfection system [43]. Prior to transfection at 1.2 × 10^6^ cells/ml, cells were diluted 1:2 with fresh culture medium and transfected with 0.4 mg/l expression plasmids and transfection reagents. Cell supernatants containing secreted proteins were typically harvested 72–96 h post-transfection. Histidine-tagged proteins were purified by immobilized metal ion affinity chromatography (IMAC). The purity and integrity of the purified proteins were analysed by RP-HPLC on an Agilent 1290 Series with a Poroshell 300SB-C8, 1 × 75 mm column (Agilent). Chromatography was performed with a non-linear (H_2_O + 0.01 % TFA/Acetonitrile + 0.08 % TFA) gradient system. The protein concentration was determined by measuring the OD_280_ (1 Abs = 1 mg/ml). The purified recombinant proteins were identified as the expected G-CyRPA and N-CyRPA proteins by western blot analysis with PfCyRPA-specific mAbs [32].

### Expression of PfCyRPA fragments on the surface of HEK cells

293 HEK cells expressing PfCyRPA fragments on the cell surface were generated essentially as described previously by Dreyer et al. [32]. Briefly, DNA sequences coding for the fragments of PfCyRPA were amplified by PCR from a plasmid containing the full length and codon-optimized sequence of PfCyRPA. The amplicons were digested with restriction endonucleases NheI and NotI (New England Biolabs) and then ligated into a pcDNA3.1-based expression vector [38]. This expression vector allows to anchor the protein of interest on the cell surface via the transmembrane domain of mouse glycophorin-A. In addition it contains the secretion signal of bee-venom melittin, a FLAG tag located extracellularly, and a hexa-His tag located in the cytosol. The 293 HEK cells were transfected with the different expression vectors using JetPEI transfection reagent (PolyPlus) according to the manufacturer’s instructions. Transient transfectants were harvested 48 h post-transfection; cell lysates were generated as described below and used for Western blot analysis.

### Immunization of mice

All procedures involving living animals were performed in strict accordance with the rules and regulations for the protection of animal rights (Tierschutzverordnung) of the Swiss Federal Food Safety and Veterinary Office. The protocol was granted ethical approval by the Veterinary Office of the county of Basel-Stadt, Switzerland (Permit numbers: 2375 and 2303). Specific pathogen-free HsdWin:NMRI outbred mice were purchased from Harlan Laboratories B.V. (The Netherlands) and used for immunizations studies. Sixteen mice were immunized intraperitoneally with 20 μg/injection of recombinant protein emulsified in aluminum hydroxide gel (Alhydrogel-2 %, Brenntag Biosector) containing CpG ODN as immune enhancer [44]. The animals received three booster injections at 2 weeks intervals with the same antigen preparation. Two weeks after the last boost, blood was collected and the serum was tested for the presence of PfCyRPA-specific antibodies by ELISA and western blot analysis.

### Fusion and cell-based selection

The best immune responders were selected for fusion. These mice received an intravenous (i.v.) injection of 20 μg of antigen dissolved in PBS 2 days before the fusion. Mice were sacrificed and the spleen was removed. Splenocytes were fused to the myeloma cell partner (PAI mouse myeloma cells, derived from SP-20, Institute of Immunology, Basel) using polyethylene glycol 1500 (Roche Diagnostics). The fusion mix was plated into 96-well culture plates and hybridomas were selected by growing in HAT-medium supplemented with culture supernatant of mouse macrophages P388. Wells were screened for IgG production 2 weeks post-fusion by ELISA as described previously [38]. IgG-producing hybrids were further screened for PfCyRPA-specific IgG production by ELISA on recombinant PfCyRPA. Positive wells were cloned in HT-medium by limiting dilution to obtain monoclonal populations.

### Antibody production and characterization

Identification of antibody subclasses was performed using a Mouse Monoclonal Antibody Isotyping Kit (ISO2, Sigma). For large-scale mAb production hybridoma cell lines were cultured in 500 ml roller-bottles (Corning). Monoclonal antibodies were purified by affinity chromatography using protein A sepharose (GE Healthcare).

### *Plasmodium falciparum* blood stage culture

*Plasmodium falciparum* strain 3D7 was cultured essentially as described previously [45]. The culture medium was supplemented with 0.5 % AlbuMAX (Life Technologies) as a substitute for human serum [46]. Cultures were synchronized by sorbitol treatment [47]. Erythrocytes for passages were obtained from the Swiss Red Cross (Switzerland). *Plasmodium falciparum* merozoites were mechanically released from mature schizonts as previously described [48]. Briefly, late-stage parasites (40–46 h post-invasion) were purified by Percoll density gradient [49] and incubated with 10 μM E-64 inhibitor (Sigma). After 6–8 h incubation, mature schizonts were filtered through 1.2 μm filters to release merozoites mechanically. Then, merozoites were resuspended PBS and stored at −80 °C until further use.

### ELISA

#### Detection of PfCyRPA-specific Abs in mouse sera by ELISA

ELISA Maxisorp plates (Nunc) were coated with 10 μg/ml purified recombinant G-CyRPA or MUL_3720 [50] proteins. After blocking, plates were incubated with dilutions of mouse serum. Horseradish Peroxidase (HRP) conjugated goat anti-mouse γ-chain specific (SouthernBiotech) was used as secondary antibody and tetramethylbenzidine substrate was used as substrate (KPL). The reaction was stopped with 0.5 M H_2_SO_4_ and the absorbance at 450 nm was measured using the Sunrise Absorbance Reader (Tecan). The cut-off value for calculation of endpoints titers was defined for each immunization group as:

$$\begin{aligned} &Average \,OD \,value\ + \left( {2 \times Standard \,Deviation \,control} \right)\end{aligned}$$

Serum IgG endpoint titers were calculated as reciprocal values of the last dilution factor yielding an OD value higher than the cut-off. Data were processed and analysed using GraphPad Prism 6.0 (GraphPad).

#### Ab competition ELISA

Plates were coated with 10 μg/ml purified recombinant G-CyRPA protein; after blocking, plates were incubated with 10, 1, or 0 μg/ml of different anti-PfCyRPA mAbs. After 30 min, different biotinylated anti-PfCyRPA mAbs were added to each well resulting in a concentration of 1 μg/ml of labelled mAb. As the two antibodies compete for the same binding site, the signal is reduced because less biotinylated detection antibody is able to bind to PfCyRPA. Alkaline phosphatase-conjugated streptavidin (Southern Biotech) was used as detecting agent, and *p*-nitrophenyl phosphate substrate (Sigma) was used for development. The OD of the reaction product was measured at 405 nm. Anti-PfCyRPA antibodies with a signal reduction higher than 30 % (compared to the absence of competitor) were considered as competing.

### Western blotting analysis

Blood stage parasite lysates were prepared essentially as described previously by saponin lysis of *P. falciparum* 3D7-infected erythrocytes [45]. In brief, cultured parasites were washed once with PBS. Pelleted infected red blood cells were lysed in 20 volumes of 0.06 % (w/v) saponin in PBS and incubated on ice for 20 min. Parasites were washed and the final pellet was resuspended in three volumes of PBS and stored at −80 °C until further use. RIPA-lysates were prepared by resuspending saponin pellets in three volumes of complete lysis buffer (1 % NP40, 0.25 % DOC, 10 % glycerol, 2 mM EDTA, 137 mM NaCl, 20 mM Tris HCl pH 8.0, protease inhibitors) for 10 min on ice. The lysates were cleared by centrifugation at 15,000*g* for 10 min and the supernatant kept at −80 °C until use. HEK cell lysate were prepared at 10^7^ cells/ml in Complete Lysis Buffer as described above. For SDS-PAGE, recombinant PfCyRPA, cell- or parasite lysates were resolved on precast 4–12 % gradient gels (NuPAGE^®^ Novex 4–12 % Bis–Tris Gel, Life Technologies) with MES running buffer according to the manufacturer’s directions. For analyses under reducing conditions, samples were reduced with 50 mM_f_ dithiothreitol (DTT) and heated to a temperature of 70 °C for 10 min prior to loading. The proteins were electrophoretically transferred to nitrocellulose membrane using a dry-blotting system (iBlot, Life Technologies). After blocking the membrane, specific proteins were detected with appropriate dilutions of mAbs followed by HRP-conjugated goat anti-mouse IgG Abs (SouthernBiotech). Blots were developed using the ECL western blotting detection reagents (Pierce).

### Immunofluorescence staining of infected erythrocytes and free merozoites

For indirect immunofluorescence microscopy, smears of infected red blood cells or free merozoites were fixed in 60 % methanol and 40 % acetone for 2 min at −20 °C, air-dried and blocked with 3 % BSA in PBS. Parasites were probed with the following antibodies: biotin-labelled anti-PfCyRPA mAb SB3.3b and Alexa 568-labelled streptavidin (Invitrogen), Alexa 488-labelled mouse anti-GAPDH 1.4a mAb [51]. The slides were mounted in mounting medium containing DAPI (ProLong Gold antifade reagent with DAPI, Life Technologies). Fluorescence microscopy was performed on a Leica DM-5000B using a 60× oil immersion objective lens and documented with a Leica DFC345FX digital camera system. Images were processed using Leica Application Suite V4 (Leica) and Adobe Photoshop^®^ CS6.

### In vitro growth inhibition assay

In vitro growth inhibition assays with *P. falciparum* strain 3D7 were conducted essentially as described [52]. Each culture was set up in triplicate in 96-well flat-bottomed culture plates. The cells were analysed in a FACSscan flow cytometer (Becton–Dickinson) using CellQuest software. A total of 30,000 cells per sample were analysed. Percent inhibition was calculated from the mean parasitaemia of triplicate test and control wells as follows:

$$Percent \, inhibition \, \left( \% \right) = \frac{control - test}{{\left( {{{control} \mathord{\left/ {\vphantom {{control} {100}}} \right. \kern-0pt} {100}}} \right)}}$$

### In vivo growth inhibition assay

Monoclonal antibodies were tested in the murine *P. falciparum* model essentially as described [32, 53]. Human blood (0.75 ml) was administered daily by the i.v. or i.p. route. Mice received a single dose of mAbs formulation by i.v. injection. The following day, mice were infected with 3 × 10^7^ parasitized erythrocytes. Parasitaemia was monitored daily by flow cytometry over 6 days (day 4–9 after mAb injection). To measure serum levels of administered mAbs, serum samples were taken 1 and 8 days after injection.

## Results

### Recombinant expression of PfCyRPA

**Fig. 1:**
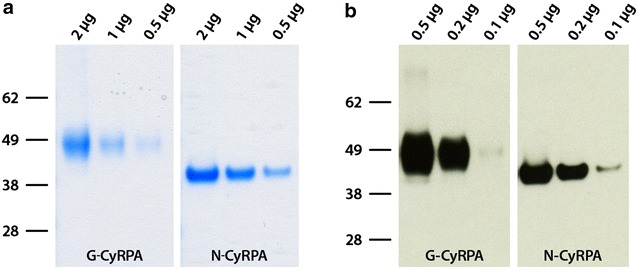
Expression of recombinant PfCyRPA. Glycosylated and non-glycosylated recombinant PfCyRPA (G-CyRPA and N-CyRPA, respectively) were expressed as His-tagged proteins in HEK cells and purified by affinity chromatography. Proteins were analysed by denaturing/reducing gel electrophoresis (SDS-PAGE) and stained with Coomassie blue (**a**), and identified by western blot analysis with the PfCyRPA-specific mAb c12 [32] (**b**)

For the production of correctly folded recombinant secreted PfCyRPA (aa. 26–362), the GPI-anchor motif was removed from the coding sequence of *PFD1130w* and expressed the ORF as hexa-Histidine (His-tag) fusion protein in the human embryonic kidney cell line FreeStyle 293-F cells. While the predicted molecular mass of the recombinant protein, designated G-CyRPA, was 40.9 kDa, a discrete band of about 48 kDa was detected both in SDS-PAGE and Western Blot analyses with a PfCyRPA-specific mAb (Fig. 1a, b). When analysed for the presence of N-glycosylation sites (NetNGlyc 1.0 Server) [Gupta et al. pers. comm], three asparagine residues were predicted as potential sites for N-glycosylation (N145, N322 and N338) in human cells. Recombinant G-CyRPA was then treated with PNGase F, which enzymatically removes N-linked carbohydrate residues from proteins. As expected, this treatment reduced the size of G-CyRPA by about 8 kDa (see Additional file 1).

The shift in molecular mass after PNGase F treatment suggests that G-CyRPA is indeed N-glycosylated when recombinantly expressed in HEK cells. Therefore, a non-glycosylated variant of PfCyRPA, designated N-CyRPA, was generated by replacing the three asparagine residue potentially implicated in N-glycosylation with glutamine residues (N145Q, N322Q, N338Q). As expected, N-CyRPA expressed in HEK cells migrated, both in SDS-PAGE and western Blot analyses with a PfCyRPA-specific mAb, as a discrete band of about 41 kDa (Fig. 1a, b; Additional file 2).

### Immunogenicity of recombinant PfCyRPA and generation of PfCyRPA-specific mAbs

**Fig. 2:**
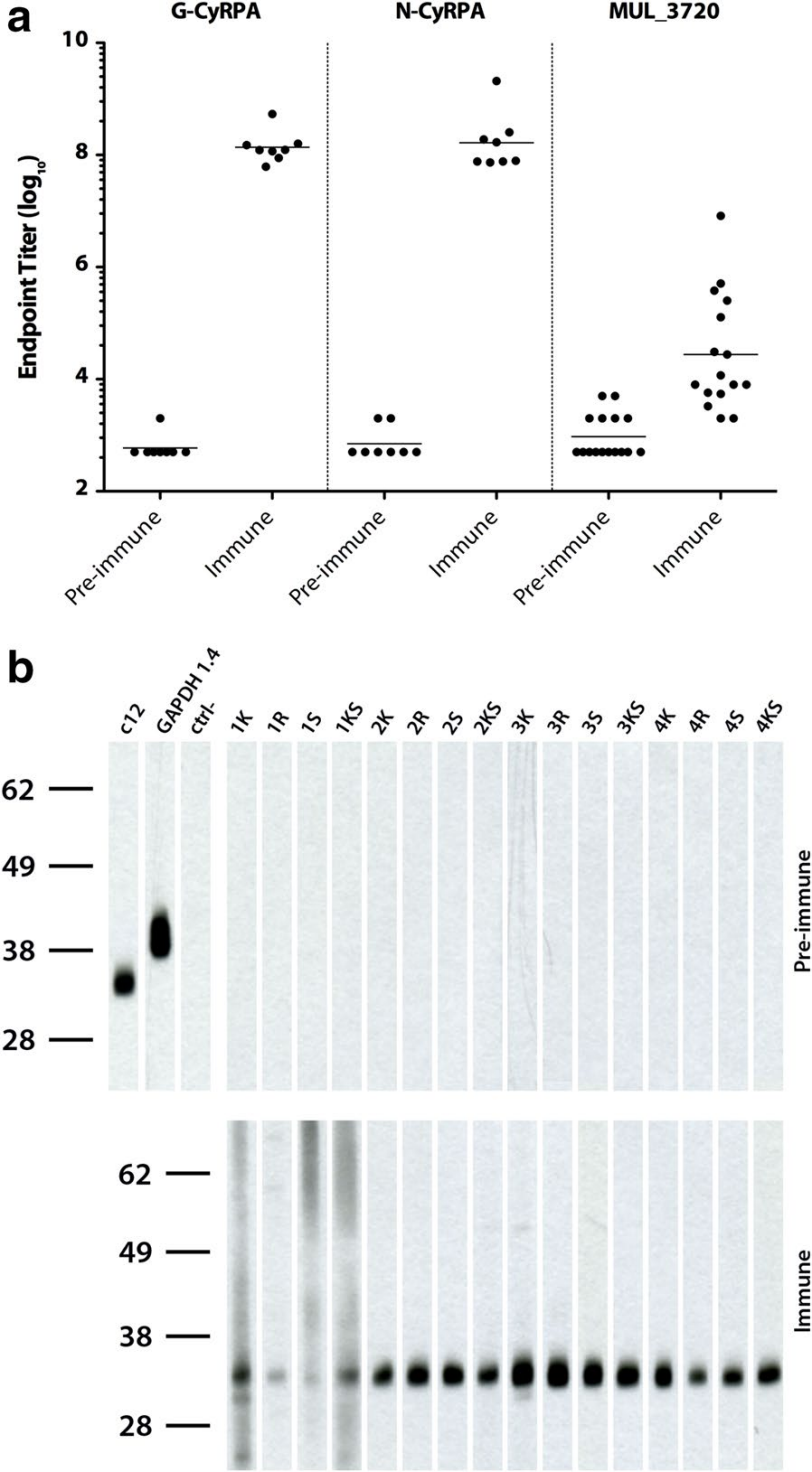
Anti-PfCyRPA IgG responses in outbred NMRI mice. Anti-PfCyRPA IgG immune responses were determined 2 weeks after the third immunization. Mice were immunized either with glycosylated (G-) or non-glycosylated (N-) recombinant PfCyRPA. **a** Serum IgG endpoint titers were measured using ELISA plates coated with recombinant G-CyRPA. As control, serum IgG endpoint titers were also measured using ELISA plates coated with an unrelated hexa-His tagged protein (MUL_3720). *Horizontal lines* designate geometric means and each data point represents one animal. Data were processed and analysed using GraphPad Prism 6.0 (GraphPad). **b** Pre-immune (*upper panel*) and immune (*lower panel*) serum samples of individual mice were tested by Western-blotting analysis on *P. falciparum* 3D7 schizont-stage parasites under non-reducing conditions. The anti-PfCyRPA mAb c12 and the anti-GAPDH mAb 1.4a were used as positive control [32, 51]; the secondary antibody without primary antibody served as negative control

**Fig. 3:**
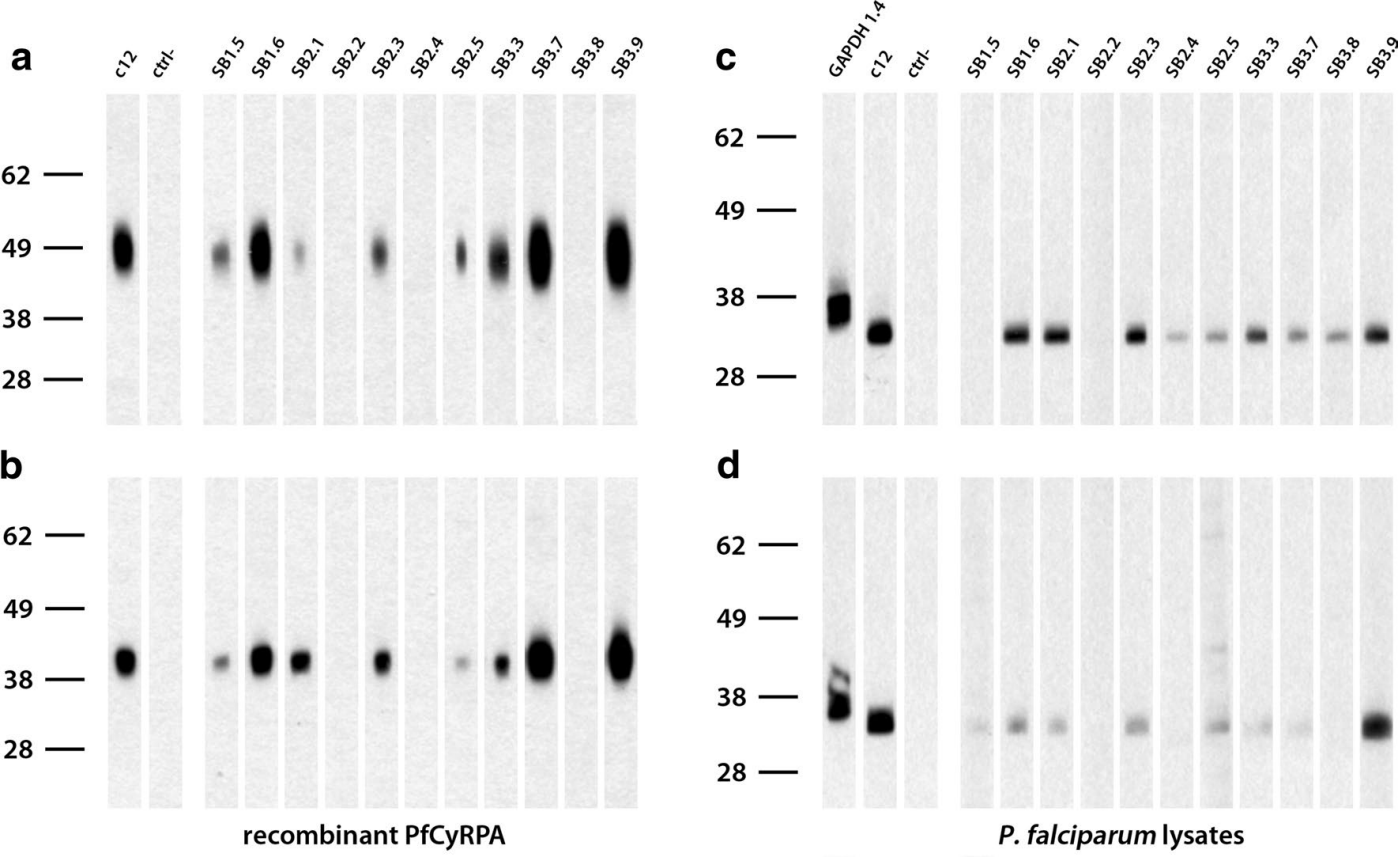
Western blotting analysis of anti-PfCyRPA mAbs. Anti-PfCyRPA mAbs were tested by western-blotting analysis on **a** recombinant G-CyRPA and **b** N-CyRPA (both sample were analysed under reducing conditions). Anti-PfCyRPA mAbs were also tested on *P. falciparum* 3D7 schizont-stage parasites under non-reducing (**c**) and reducing conditions (**d**). The anti-PfCyRPA mAb c12 and the anti-GAPDH mAb 1.4a were used as positive control [32, 51]; the secondary antibody without primary antibody served as negative control

**Fig. 4:**
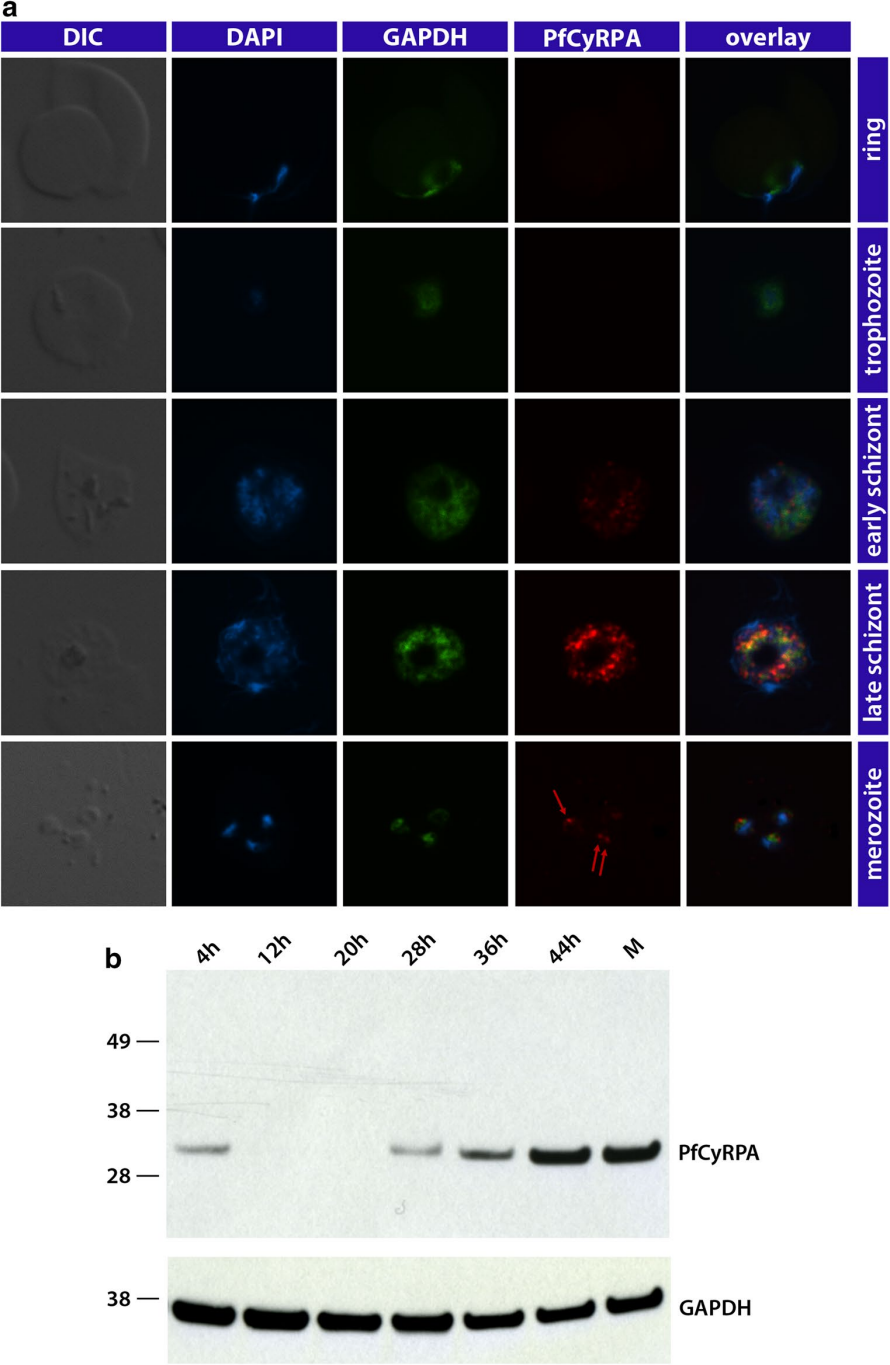
Stage specific expression of PfCyRPA in late asexual blood stage parasites. **a** Indirect immunofluorescence staining of synchronized asexual blood stage parasites showed stage specific expression of PfCyRPA in schizont stages and free merozoites. Methanol/acetone fixed *P. falciparum* 3D7 parasites were probed with the anti-GAPDH mAb (*green*) and the anti-PfCyRPA mAb SB3.3 (*red*) as representative example. Nuclei were stained with DAPI (*blue*). Exposure times were identical for all pictures of the same channel. **b** Western blot analysis with lysates of tightly synchronized *P. falciparum* 3D7 blood-stage parasites with anti-PfCyRPA mAb SB1.6 (*upper panel*). The blot was probed for equal loading with an anti-GAPDH 1.4a mAb (*lower panel*). 4, 12, 20, 28, 36, 44 h post-invasion; *M* free merozoites

Mice were immunized three times with 20 µg of recombinant G-CyRPA or N-CyRPA emulsified in aluminium hydroxide containing CpG ODN as immune enhancer [44]. After the third immunization, PfCyRPA-specific serum IgG endpoint titers were determined by indirect ELISA. All mice raised antibody responses against recombinant PfCyRPA, irrespective of the immunogen’s glycosylation state (P = 0.9591, Mann-Withney test, 95 % confidence interval, two-tailed P value). As expected, mice raised also marginal antibody responses against the hexa-His tag, as indicated by a low antibody titer against the hexa-His tagged irrelevant protein MUL_3720 (Fig. 2a). Also, all immunized mice raised immune responses cross-reactive with *P. falciparum* endogenous PfCyRPA, as shown by western blot analyses on late blood-stage parasite lysates (Fig. 2b). Hybridoma cell lines producing PfCyRPA-specific mAbs were generated by fusing splenocytes of immunized mice with myeloma cells. Based on reactivity to recombinant PfCyRPA in ELISA, a panel of 11 anti-PfCyRPA mAbs was generated and tested for reactivity on recombinant and *P. falciparum* expressed PfCyRPA by western blot analyses. 8/11 anti-PfCyRPA mAbs stained the recombinant PfCyRPA band and ten of them also stained either strongly (mAbs SB1.6, SB2.1, SB2.3, SB3.3 and SB3.9) or weakly (SB1.5, SB2.4, SB2.5, SB3.8 and SB3.8) a band of the size expected for PfCyRPA in *P. falciparum* schizont-stage lysate (Fig. 3; Table 1). In indirect immunofluorescence staining of asexual blood stage parasites, 9/11 mAbs were positive. mAb SB2.2 was positive in ELISA with recombinant PfCyRPA, but negative both in western blot analysis and in IFA (Table 1). With the exception of mAb SB3.8, which in ELISA was reacting only to recombinant G-CyRPA, no general differences in binding properties, between anti-PfCyRPA mAbs produced against glycosylated or non-glycosylated recombinant PfCyRPA, were observed (Table 1). In indirect immunofluorescence and western blotting analyses mAbs stained asexual blood stage parasites stage-specifically (Fig. 4; Table 1), since PfCyRPA is only expressed by schizonts and free merozoites [32]. As expected, the generated mAbs showed the characteristic pattern [32] consisting of a small spot toward the merozoite apical pole (see Additional file 3).

**Table 1:** Summary of anti-PfCyRPA mAbs characterization

mAb	Immunization	ELISA		Western blotting analysis			IFA	GIA	Epitope group
		G-CyRPA	N-CyRPA	G-CyRPA	N-CyRPA	*P. falc*. 3D7	*P. falc*. 3D7	*P. falc*. 3D7	
SB1.5	G-CyRPA	+	+	+	+	+	−	Partial	nd
SB1.6	G-CyRPA	+	+	+	+	+	+	Yes	F
SB2.1	N-CyRPA	+	+	+	+	+	+	Yes	F
SB2.2	N-CyRPA	+	+	−	−	−	−	Partial	nd
SB2.3	N-CyRPA	+	+	+	+	+	+	Yes	F
SB2.4	N-CyRPA	+	+	−	−	+	+	No	nd
SB2.5	N-CyRPA	+	+	+	+	+	+	Partial	A
SB3.3	G-CyRPA	+	+	+	+	+	+	Yes	F
SB3.7	G-CyRPA	+	+	+	+	+	+	Partial	B
SB3.8	G-CyRPA	+	−	−	−	+	+	No	nd
SB3.9	G-CyRPA	+	+	+	+	+	+	No	E

Anti-PfCyRPA mAbs were characterized by ELISA (+, OD value higher than control anti-6xHis mAb HIS-6/9; −, OD value ≤ control mAb), western blotting analyses (+, staining; −, no staining; cf. Fig. 3), immunofluorescence assays (+, staining; −, no staining), growth inhibition assays (yes, GIA ≥40 %; no, GIA ≤20 %) and categorized into distinct epitope groups by epitope mapping experiments (cf. Fig. 8). G-CyRPA designates recombinant glycosylated PfCyRPA whereas N-CyRPA designates non-glycosylated recombinant PfCyRPA

### Vaccine elicited anti-PfCyRPA mAbs inhibit parasite growth in vitro

**Fig. 5:**
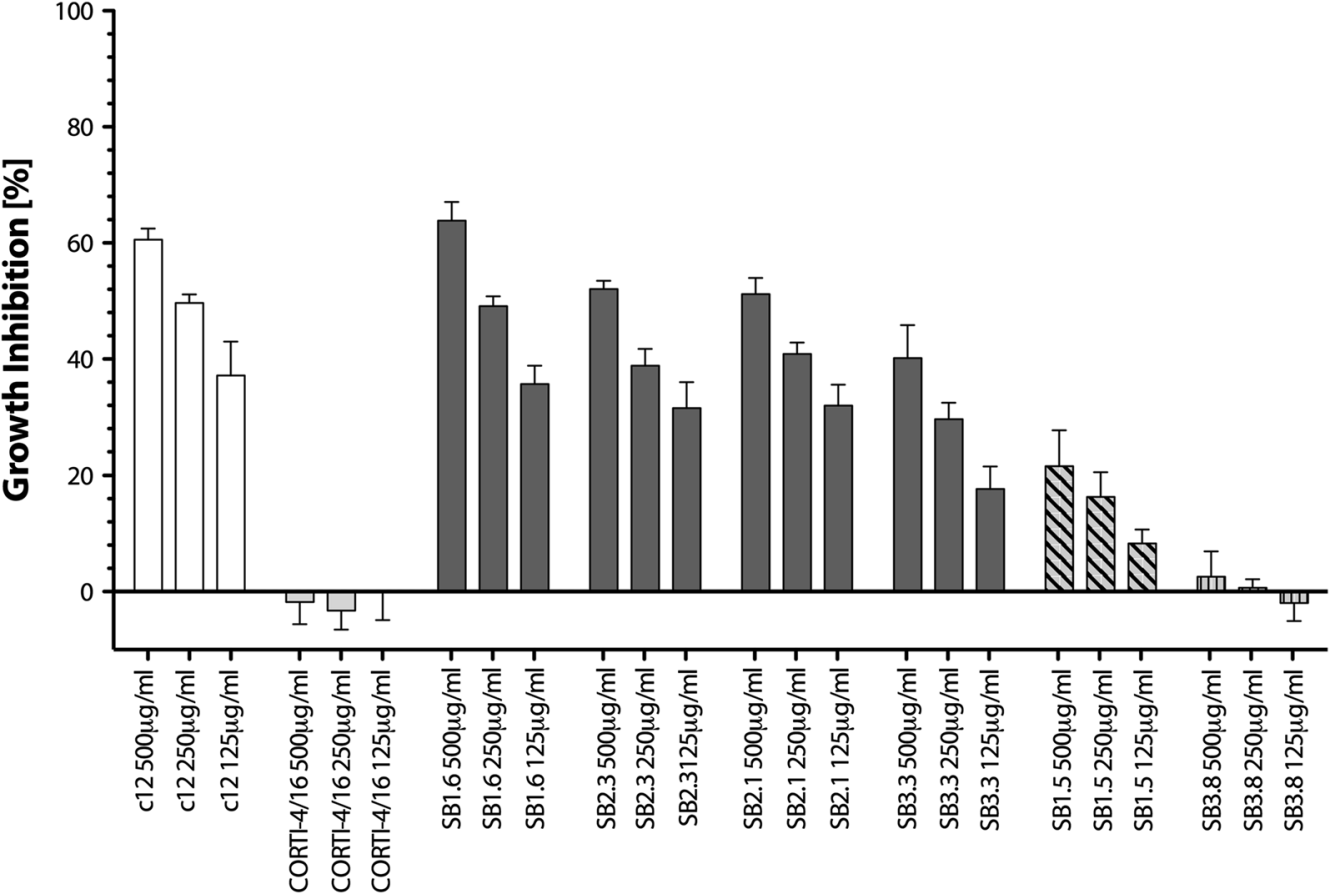
Vaccine elicited anti-PfCyRPA mAbs inhibit parasite growth in vitro. Synchronized *P. falciparum* 3D7 parasites were cultivated for 96 h in the presence of anti-PfCyRPA mAbs. The percentage of growth inhibition was calculated against the parasitaemia of PBS control wells. *Each bar* represents the mean of a triplicate experiment, and *error bars* indicate the SD. Anti-PfCyRPA mAbs SB1.6, SB2.3, SB2.1 and SB3.3 are growth inhibitory mAbs, mAb SB1.5 is shown as an example for a partially inhibitory mAb, and mAb SB3.8 as an example for a non-inhibitory mAb. The anti-cortisol mAb CORTI-4/16 was used as negative control and the anti-PfCyRPA mAb c12, which was produced after immunization with mammalian cells expressing recombinant PfCyRPA on their cell surface, as positive control [32]

For the analysis of the growth inhibitory activity of mAbs generated against the purified recombinant PfCyRPA, parasites were cultured for two cycles of merozoite invasion in the presence of anti-PfCyRPA mAbs at concentrations of 500, 250, and 125 µg/ml. Anti-PfCyRPA mAbs with parasite growth inhibitory activity higher than 40 % at the concentration of 500 µg/ml were classified as inhibitory and those with an inhibitory effect ranging between 20 and 40 %, were classified as partially inhibitory. Among the four inhibitory mAbs (SB1.6, SB2.1, SB2.3, and SB3.3), SB1.6 showed the highest activity by reducing parasite growth by 64 ± 3.2 % when tested at a concentration of 500 µg/ml (Fig. 5; Table 1). Another four anti-PfCyRPA mAbs inhibited parasite growth partially, whereas three mAbs showed no inhibitory effect (Fig. 5; Table 1). All tested mAbs were produced and purified in the same way and results were reproducible in independent experiments and with independent mAb production batches (see Additional file 4).

### Vaccine elicited anti-PfCyRPA mAbs inhibit parasite growth in vivo

**Fig. 6:**
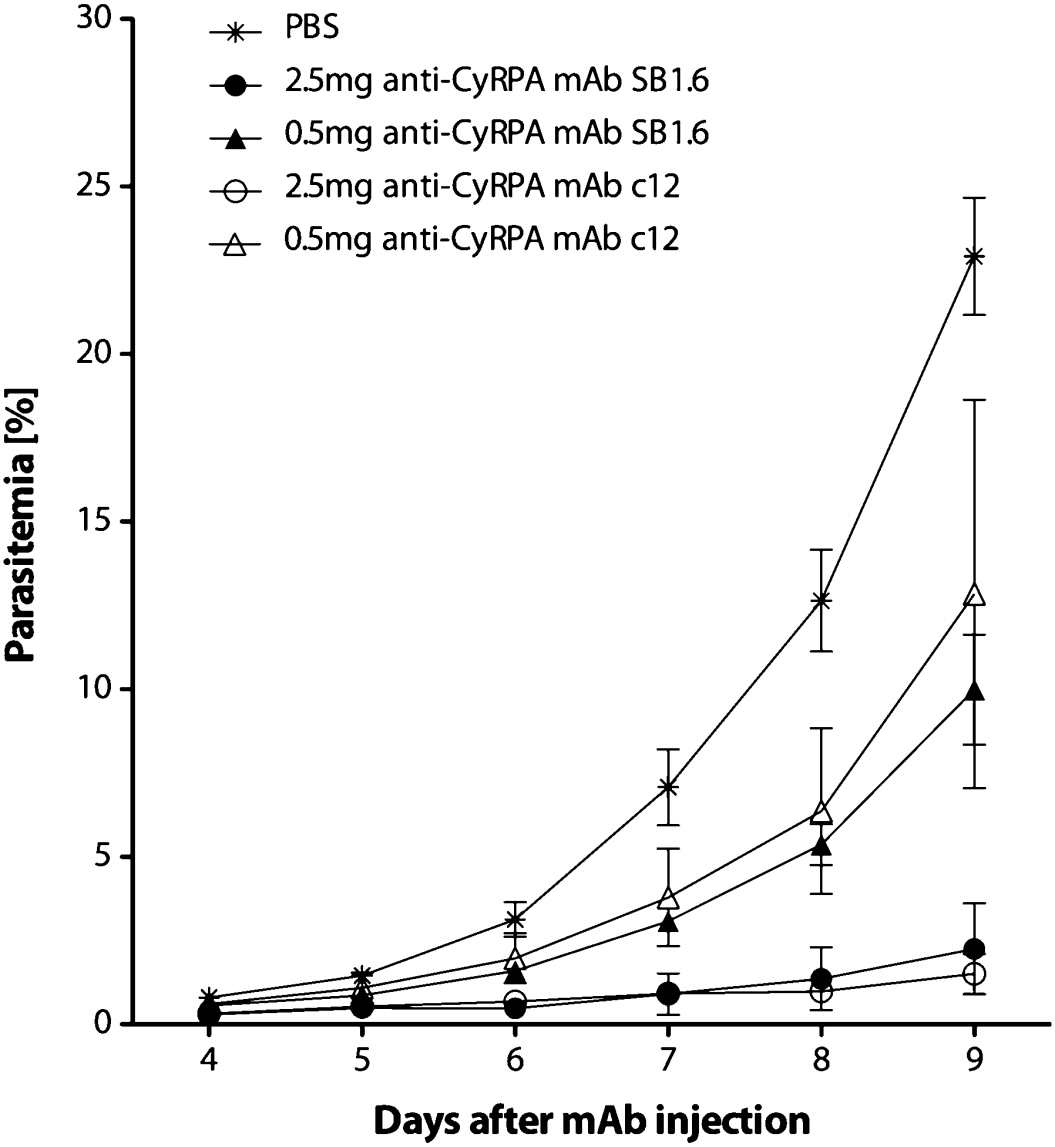
Vaccine elicited anti-PfCyRPA mAbs inhibit parasite growth in vivo. NOD-*scid IL2Rγ* ^*null*^ mice received purified anti-PfCyRPA mAbs by i.v. injection. Mice were then infected with *P. falciparum* 3D7 and parasitaemia was monitored over 6 days. Values are the mean parasitaemia in human erythrocytes in peripheral blood of three mice per group. *Error bars* indicate the SD. PBS was used as negative control, the anti-PfCyRPA mAb c12 as positive control [32]

The in vivo parasite inhibitory activity of the generated anti-PfCyRPA mAbs was evaluated in the *P. falciparum* SCID murine model [53] that employs non-myelodepleted NOD-scid *IL2Rγ*^*null*^ mice engrafted with human erythrocytes. Groups of three mice received 2.5 or 0.5 mg of PfCyRPA-specific mAbs, either mAb SB1.6 generated here, or the previously described [32] invasion-inhibitory mAb c12, by i.v. injection. The control group received the same volume of PBS. Mice were infected with parasitized erythrocytes 1 day after the antibody transfer and parasitaemia of all mice was monitored for 6 days (Fig. 6). In the control group, parasitaemia reached 22.9 ± 1.7 % on day 9. Parasitaemia in mice having received 2.5 mg anti-PfCyRPA mAbs SB1.6 or c12 increased only marginally, reaching 2.2 ± 1.4 and 1.5 ± 0.6 % on day 9 after mAb injection, respectively. At the lower dose of 0.5 mg anti-PfCyRPA mAbs SB1.6 or c12 inhibited parasite growth to 10 ± 1.6 and 12.8 ± 5.8 % parasitaemia, respectively (Fig. 6). Titration of administered mAbs in the serum of the passively immunized mice by ELISA showed that antibodies, although at reduced level, persisted over the entire study period. One day after mAb injection (2.5 mg dose), when mice were infected, PfCyRPA-specific mAb concentrations were estimated to be 295 ± 0.3 and 389 ± 0.1 % μg/ml of serum, for SB1.6 and c12, respectively. Eight days after mAb injection, at the end of the experiment, antibody concentrations had dropped down to 74 ± 0.1 and 83 ± 0.2 % μg/ml, for SB1.6 and c12, respectively (see Additional file 5).

### Fine specificities of anti-PfCyRPA mAbs

**Fig. 7:**
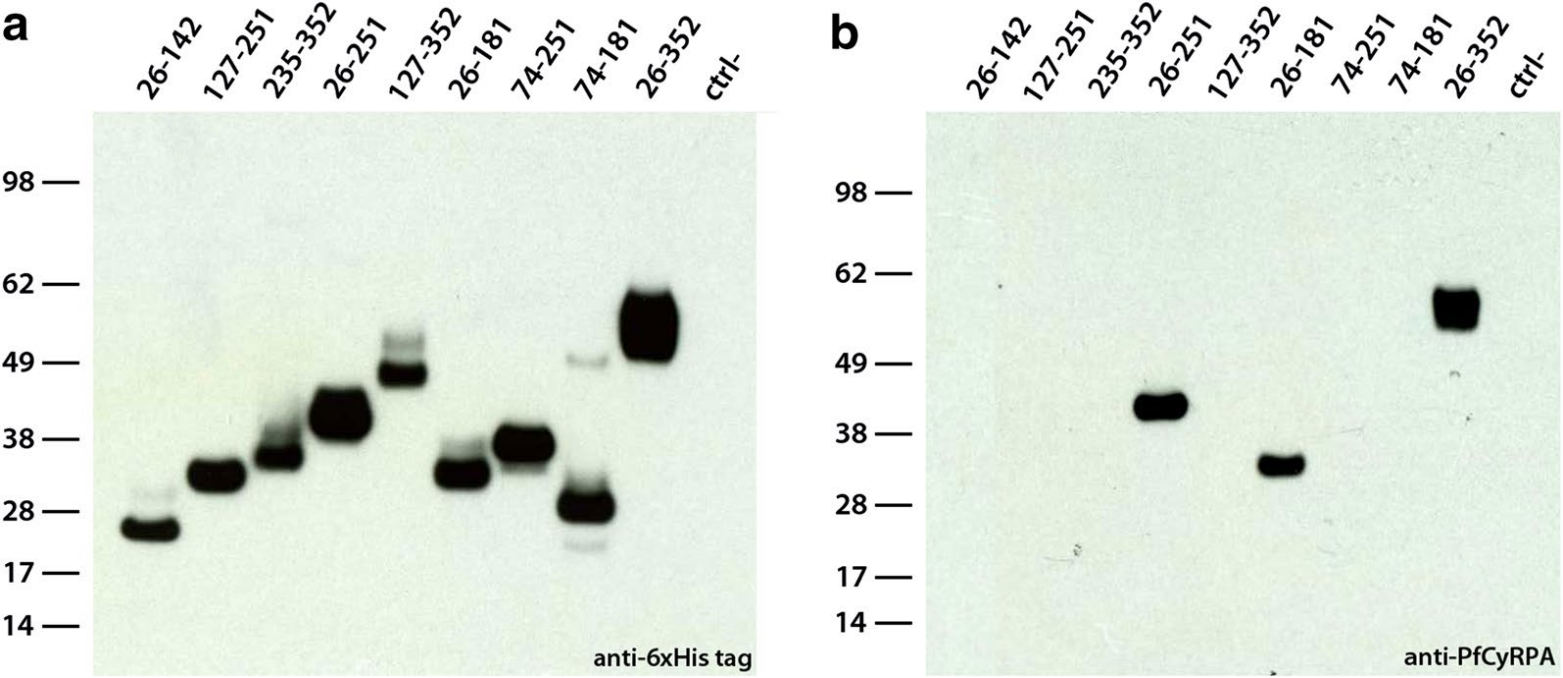
Reactivity pattern of anti-PfCyRPA mAbs with recombinant PfCyRPA fragments. Anti-PfCyRPA mAbs were tested by western blot analysis on lysates of HEK cells expressing recombinant PfCyRPA fragments. A lysate HEK cells transfected with empty plasmids served as negative control. Both samples were analysed under reducing conditions. **a** Reactivity pattern of anti-6xHis mAb HIS-6/9 as positive control for expression. **b** Reactivity pattern of anti-PfCyRPA mAb SB 3.7 as a representative example

**Fig. 8:**
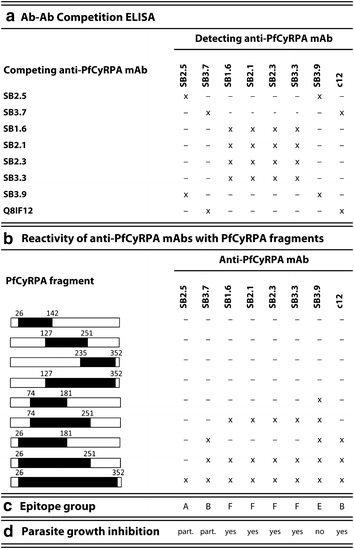
Epitope specificities of anti-PfCyRPA mAbs. **a** Anti-PfCyRPA mAbs were tested in an Ab–Ab competition ELISA. *x* Ab competition (signal reduction higher than 30 %; measured values: min. 34 %, max. 91 % reduction), *−* no Ab competition (measured values: min. −10 %, max. 21 % reduction. **b** Reactivity of anti-PfCyRPA mAbs with recombinant PfCyRPA fragments were investigated by western blotting analyses performed on lysates of HEK cells expressing recombinant PfCyRPA fragments. *x* staining, *−* no staining (an example of the reactivity pattern is reported in Fig. 7b). **c** Anti-PfCyRPA mAbs were assigned to different epitope groups according to the results from the Ab–Ab competition ELISA and the epitope mapping experiment. **d** *P. falciparum* in vitro growth inhibition activity of mAbs

The fine specificities of the generated anti-PfCyRPA mAbs were analysed by Ab–Ab competition ELISA on recombinant PfCyRPA. The in vitro growth-inhibitory anti-PfCyRPA mAbs SB1.6, SB2.1, SB2.3, and SB3.3 competed against each other for their antigen binding site (Fig. 8a). These four mAbs did neither compete with the partially inhibitory mAbs SB2.5 and SB3.7 nor with the previously described parasite inhibitory anti-PfCyRPA mAb c12. Additionally, the reactivity of anti-PfCyRPA mAbs with overlapping protein fragments of PfCyRPA [32] was tested by western blotting analysis (Figs. 7, 8b). With the exception of SB2.5, all tested anti-PfCyRPA mAbs reacted with fragment 26–251. All four inhibitory anti-PfCyRPA mAbs SB1.6, SB2.1, SB2.3, and SB3.3 recognized the same fragments, i.e., bound to fragment 74–251, but not to the truncated sub-fragments 26–142, 74–181, and 127–251, indicating that they recognize a conformational epitope present in fragment 74–251. Furthermore, the partially inhibitory mAbs SB3.7 and the control mAb c12 showed the same reactivity pattern. Based on the complementary results of both epitope classification assays, the anti-PfCyRPA mAbs was assigned to four distinct epitope groups (Fig. 8b, c).

## Discussion

On the basis of available genome-wide transcriptomic and proteomic data, Dreyer et al. [38] have selected uncharacterized surface proteins, with specific expression in extracellular parasite stages, to evaluate their potential as blood stage vaccine candidate antigens. A panel of candidates was characterized (e.g., abundance, distribution and parasite growth inhibitory potential) using antigen-specific mAbs, which were generated exploiting a cell-based approach that utilizes antigen-expressing living cells for mouse immunization. This strategy has led to the identification of PfCyRPA as promising blood-stage malaria vaccine candidate: generated anti-PfCyRPA mAbs showed parasite in vitro and in vivo growth-inhibitory activity due to inhibition of merozoite invasion [32]. Since antigen-loaded mammalian cells are not suitable for human immunization, here it was investigated whether growth inhibitory anti-PfCyRPA Abs could be raised by active immunization with adjuvanted purified recombinant PfCyRPA protein.

The expression of *Plasmodium* antigens in heterologous hosts as stable recombinant protein can be challenging. Since PfCyRPA is a cysteine-rich protein, disulfide bonds play an important role in its folding. Aiming at the production of a properly folded recombinant protein, a eukaryotic rather than a prokaryotic expression system was used. Since PfCyRPA was successfully expressed in a native conformation on the surface of HEK cells and raised parasites-cross reactive mAbs [32], the same mammalian expression platform was exploited for the production of secreted PfCyRPA. For this purpose, the expression plasmid coding for PfCyRPA was modified to produce the secreted version of the protein by removing the sequence coding for the transmembrane domain artificially used to display the protein on the cell surface. PfCyRPA was expressed and secreted into the cultivation medium in good quality and quantity (ca.18 mg/l), and the glycosylated recombinant protein was easily purified via the hexa-His tag. Since the protein glycosylation status may influence immunogenicity, a non-glycosylated version of PfCyRPA was also produced, but no marked difference between the two proteins could be found with respect to their immunogenicity. To dissect and characterize the properties of the elicited anti-PfCyRPA antibody response, mice immunized with either the glycosylated or the non-glycosylated recombinant protein were employed to generate a panel of eleven IgG mAbs reactive with recombinant PfCyRPA in ELISA. Nine of the generated mAbs were cross-reacting in indirect immunofluorescence analysis with *P. falciparum* asexual blood stage parasites, yielding a dotted staining pattern characteristic for PfCyRPA and ten of them stained a band of the size expected for PfCyRPA in western blotting analysis with *P. falciparum* schizont stage lysate. Four mAbs showed strong and another four partial parasite blood stage in vitro growth inhibitory activity. The parasite inhibitory activity of mAb SB1.6 showing the strongest in vitro activity was comparable to that of the previously described anti-PfCyRPA mAb c12 which was produced after immunization with mammalian cells expressing recombinant PfCyRPA on their cell surface [32]. The strongly growth inhibitory mAbs c12 and SB1.6 do not compete for antigen binding (Fig. 8), confirming that PfCyRPA harbours more than one target epitope for inhibitory antibodies, as already suggested by Dreyer et al. [32].

Structural analyses with antigen–antibody complexes are required to gain deeper insight into the targets and mode of action of these antibodies. Since orthologs of PfCyRPA are only present in the human malaria parasite *P. vivax* and the primate pathogens *P. knowlesi*, *P. cynomolgi*, and *P. reichenowi* [54–56], but are absent in *Plasmodium* species infecting rodents, conventional mouse models with rodent parasites cannot be used to evaluate the in vivo growth inhibitory activity of anti-PfCyRPA mAbs. Therefore, passive immunization experiments were performed exploiting an innovative *P. falciparum* SCID mouse model [32, 53].

Non myelo-depleted NOD-*scid IL2Rγ*^*null*^ mice, engrafted with human erythrocytes to allow the growth of *P. falciparum,* received a single dose of anti-PfCyRPA mAbs via the i.v. route and were infected with *P. falciparum* 3D7 parasites on the subsequent day. In mice receiving SB1.6 mAb, a strong, dose-dependent parasite growth inhibitory effect was observed, reducing parasite’s growth by about 90 % (2.5 mg dose). The concentration of mAb SB1.6 in the circulation of the passively immunized mice which received the higher dose, was estimated to be 300 and 80 μg/ml 1 and 8 days after injection, respectively. Since SCID mice lack the adaptive immune system and have deficiencies in the innate immune system [57, 58], the injected mAbs were the only circulating IgG, and this may enhance their clearance from the circulation. However, the measured serum concentration of PfCyRPA-specific antibody fall in the range of specific Abs that can be induced by appropriate vaccine formulations [8, 37]. In this context, it should also be taken into account that immunizations with recombinant PfCyRPA (both antigen-loaded cells and adjuvanted purified proteins) generated anti-PfCyRPA mAbs with different fine specificity [32].

Hence, stronger inhibitory activities may be achieved in the context of active immunizations, where Abs specific for more than one inhibitory epitope are induced, and lower titers of total PfCyRPA-specific Abs may be required to confer substantial protection. As already described [32], anti-PfCyRPA mAbs reduce, but do not completely block, parasite growth by inhibiting a crucial invasion pathway of erythrocytes by merozoites.

Invasion of host erythrocytes is a complex and critical step in the life cycle of malaria parasites, and *P. falciparum* has evolved an abundance of antigenically diverse, and probably functionally redundant, merozoite surface proteins to facilitate parasite escape from host immune detection and ensure invasion via multiple pathways [59, 60]. In this respect, marginal sequence polymorphisms and limited natural immunogenicity of PfCyRPA [32] suggest a critical function of PfCyRPA in erythrocytes invasion, which prevents sequence variation and accessibility to the immune system in the natural context. PfCyRPA has been recently identified as the anchor protein that tethers PfRH5, and its interacting partner PfRipr, to the surface of merozoites [33]. PfRH5 has been shown to play a key role in the attachment of merozoites to the erythrocyte surface via the interaction with the host receptor basigin [61, 62]. Interestingly, PfCyRPA and PfRH5 genes are located in close proximity in the genome, have no substantial sequence polymorphisms, have demonstrated poor natural immunogenicity, and elicit potent and strain-transcending growth-inhibitory parasite antibodies [32, 34]. Anti-PfCyRPA mAb concentrations required for substantial growth inhibition in GIA were higher than those reported for anti-basigin (1 μg/ml) and anti-PfRH5 (15 μg/ml) mAbs, respectively [36]. This may be in part related to different assay formats, but also to other factors, such as accessibility of the antigens and kinetic and thermodynamic features of mAb binding. Reddy et al. [33] also reported a synergistic in vitro inhibitory activity for the combination of polyclonal anti-PfCyRPA and anti-PfRH5 antibodies. Targeting simultaneously PfCyRPA and PfRH5 seems to hinder parasite invasion more effectively than when blocking only one component of the multiprotein invasion complex. Taken together, these findings suggest that additional investigation are needed for an in depth characterization of the invasion complex, and make both PfCyRPA and PfRH5 appealing candidates for the development of new anti-malarial vaccine strategies.

## Conclusions

A vaccine formulation composed of adjuvanted recombinantly expressed PfCyRPA has been shown to elicit in mice high titers of antibodies that inhibit both in vitro and in vivo *P. falciparum* asexual blood stage parasite growth. These findings qualify PfCyRPA, a highly conserved and poorly immunogenic merozoite protein, as highly suitable candidate antigen for inclusion into a strain-transcending, multivalent malaria subunit vaccine.

## Additional files

10.1186/s12936-016-1213-x PNGase F treatment of recombinant G-CyRPA. 1 mg/ml of native (second lane) or reduced with 50mM_f_ DTT (third lane) G-CyRPA was subjected for 16 h at room temperature to PNGase F at a final concentration of 0.05 mg/ml, and analysed by reducing SDS-PAGE (1 μg protein per lane). Lane 1: untreated G-CyRPA; lane 4: PNGase F.
10.1186/s12936-016-1213-x Recognition of N-CyRPA by PfCyRPA- and 6xHis-specific mAbs. Reducing (R) and non-reducing (N) SDS-PAGE of non-glycosylated PfCyRPA (N-CyRPA) detected by Coomassie-staining (blue) and Western blotting with anti-PfCyRPA mAb c12 and anti-6xHis mAb HIS-6/9 (black). Analyses under non-reducing conditions revealed both a monomeric and a dimeric form of PfCyRPA.
10.1186/s12936-016-1213-x Localization of PfCyRPA in late asexual blood stage parasites. Indirect immunofluorescence staining of *P. falciparum* 3D7 schizont stages. Methanol/acetone fixed parasites were co-immunostained with mAbs against PfCyRPA (red) and AMA-1 (marker for micronemes), RAP-1 (marker for rhoptry bulbs), MSP-1 or MSP-5 (marker for merozoite’s surface) (green). Parasites were probed with the following primary or secondary antibodies: biotin-labeled anti-PfCyRPA mAb SB3.3b, Alexa 488-labeled mouse anti–AMA-1 DV5a mAb [52], Alexa 488-labelled mouse anti–RAP-1 5-2 mAb [63], anti–MSP-1 MC7.2 mAb (G. Pluschke, unpublished), anti–MSP-5 rabbit serum (MRA-320; Malaria Research and Reference Reagent Resource Center) [64], Alexa 568-labeled streptavidin (Invitrogen), Alexa 488-labelled goat anti-mouse IgG (H + L) Abs, and Alexa 488-labeled chicken anti-rabbit IgG (H + L) Abs (Invitrogen). Nuclei were stained with DAPI (blue). Exposure times were identical for all pictures of the same channel.
10.1186/s12936-016-1213-x *In vitro* parasite growth inhibitory activity of vaccine elicited anti-PfCyRPA mAbs was reproducible in independent experiments. Different batches of anti-PfCyRPA mAbs were produced and purified in the same way, and tested in independent in vitro growth inhibition assays. Reported are three independent experiments as representative examples of obtained results. Anti-PfCyRPA mAbs SB1.6 is shown as an example for an inhibitory mAb, mAb SB1.5 for a partially inhibitory mAb, and mAb SB3.8 for a non-inhibitory mAb. The anti-cortisol mAb CORTI-4/16 was used as negative control and the anti-PfCyRPA mAb c12 as positive control [32]. Each bar represents the mean of a triplicate experiment, and error bars indicate the SD.
10.1186/s12936-016-1213-x Titration of administered mAbs in the serum of the passively immunized mice. The concentration of administered PfCyRPA-specific mAbs SB1.6 and c12 in the circulation was estimated by indirect ELISA on day one and eight after injection. For the detection of PfCyRPA-specific mAbs and total circulating IgGs, plates were coated with N-CyRPA or goat anti-mouse IgG (γ-chain specific) mAb (M1397, Sigma), respectively. After blocking, plates were incubated with dilutions of individual mouse serum. An HRP-conjugated goat anti-mouse IgG (γ-chain specific) Ab (A3673, Sigma) was used as secondary antibody and TMB as substrate. Standard curves were generated from known dilutions of SB1.6 and c12 mAbs and fit using a 4-PL logistic equation. Concentration of circulating mAbs was calculated by interpolating the absorbance values for the test sera from the standard curves. Reported values are means of three mice per group ± SD.
