# Supplementary figures and images for: Generation of Plasmodium falciparum parasite-inhibitory antibodies by immunization with recombinantly-expressed CyRPA
Source: Malar J. 2016 Mar 15;15:161. doi: 10.1186/s12936-016-1213-x (PMC4791974; doi:10.1186/s12936-016-1213-x)

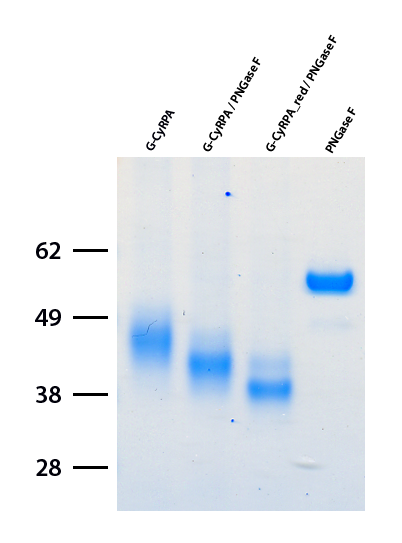

Supplement: Supplementary file 1 — 10.1186/s12936-016-1213-x PNGase F treatment of recombinant G-CyRPA. 1 mg/ml of native (second lane) or reduced with 50mMf DTT (third lane) G-CyRPA was subjected for 16 h at room temperature to PNGase F at a final concentration of 0.05 mg/ml, and analysed by reducing SDS-PAGE (1 μg protein per lane). Lane 1: untreated G-CyRPA; lane 4: PNGase F. [file 12936_2016_1213_MOESM1_ESM.tif]

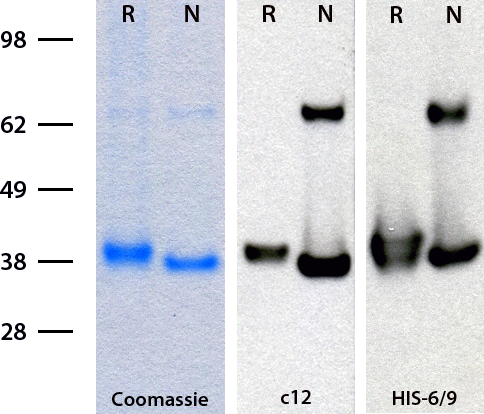

Supplement: Supplementary file 2 — 10.1186/s12936-016-1213-x Recognition of N-CyRPA by PfCyRPA- and 6xHis-specific mAbs. Reducing (R) and non-reducing (N) SDS-PAGE of non-glycosylated PfCyRPA (N-CyRPA) detected by Coomassie-staining (blue) and Western blotting with anti-PfCyRPA mAb c12 and anti-6xHis mAb HIS-6/9 (black). Analyses under non-reducing conditions revealed both a monomeric and a dimeric form of PfCyRPA. [file 12936_2016_1213_MOESM2_ESM.tif]

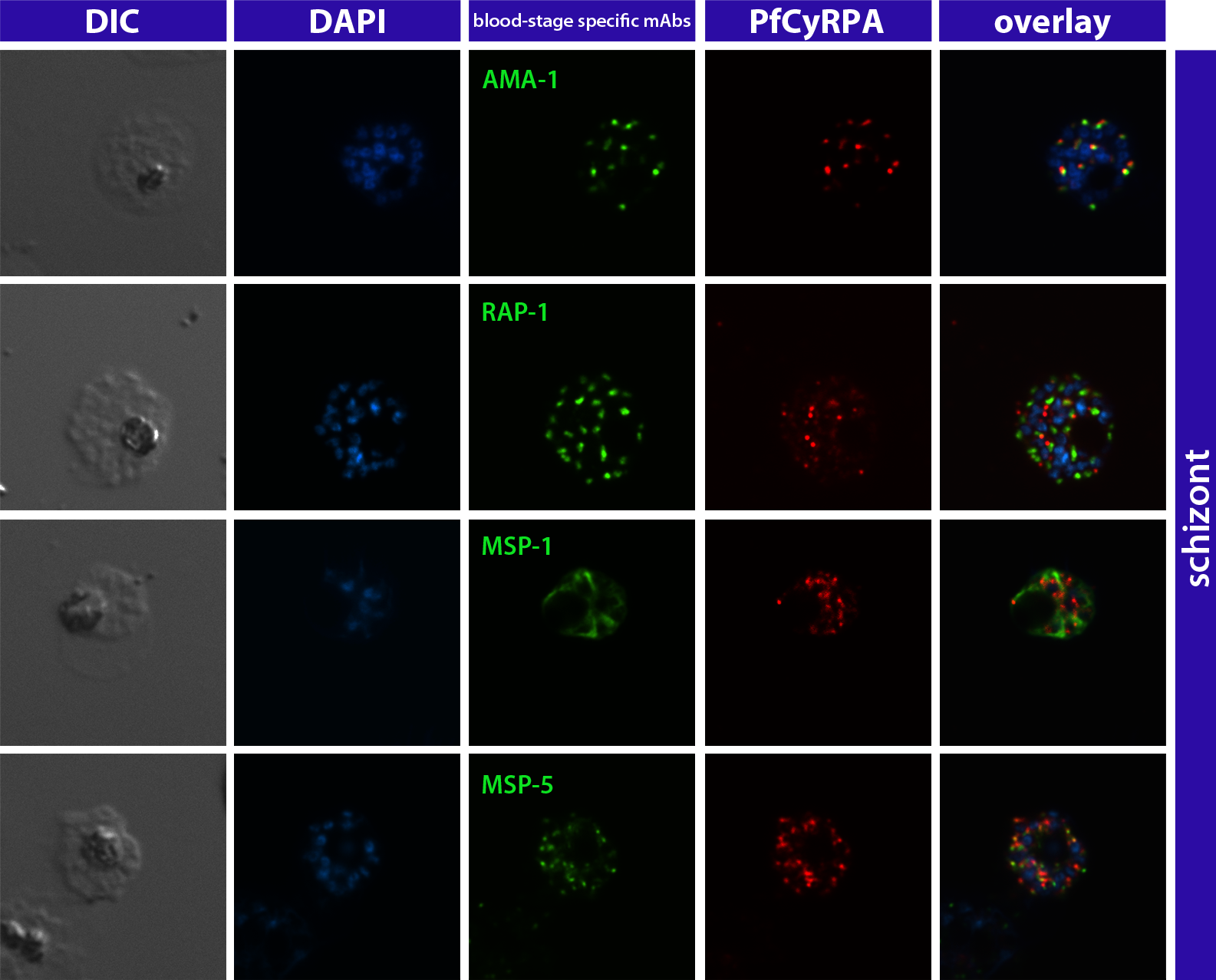

Supplement: Supplementary file 3 — 10.1186/s12936-016-1213-x Localization of PfCyRPA in late asexual blood stage parasites. Indirect immunofluorescence staining of P. falciparum 3D7 schizont stages. Methanol/acetone fixed parasites were co-immunostained with mAbs against PfCyRPA (red) and AMA-1 (marker for micronemes), RAP-1 (marker for rhoptry bulbs), MSP-1 or MSP-5 (marker for merozoite’s surface) (green). Parasites were probed with the following primary or secondary antibodies: biotin-labeled anti-PfCyRPA mAb SB3.3b, Alexa 488-labeled mouse anti–AMA-1 DV5a mAb [52], Alexa 488-labelled mouse anti–RAP-1 5-2 mAb [63], anti–MSP-1 MC7.2 mAb (G. Pluschke, unpublished), anti–MSP-5 rabbit serum (MRA-320; Malaria Research and Reference Reagent Resource Center) [64], Alexa 568-labeled streptavidin (Invitrogen), Alexa 488-labelled goat anti-mouse IgG (H + L) Abs, and Alexa 488-labeled chicken anti-rabbit IgG (H + L) Abs (Invitrogen). Nuclei were stained with DAPI (blue). Exposure times were identical for all pictures of the same channel. [file 12936_2016_1213_MOESM3_ESM.tif]

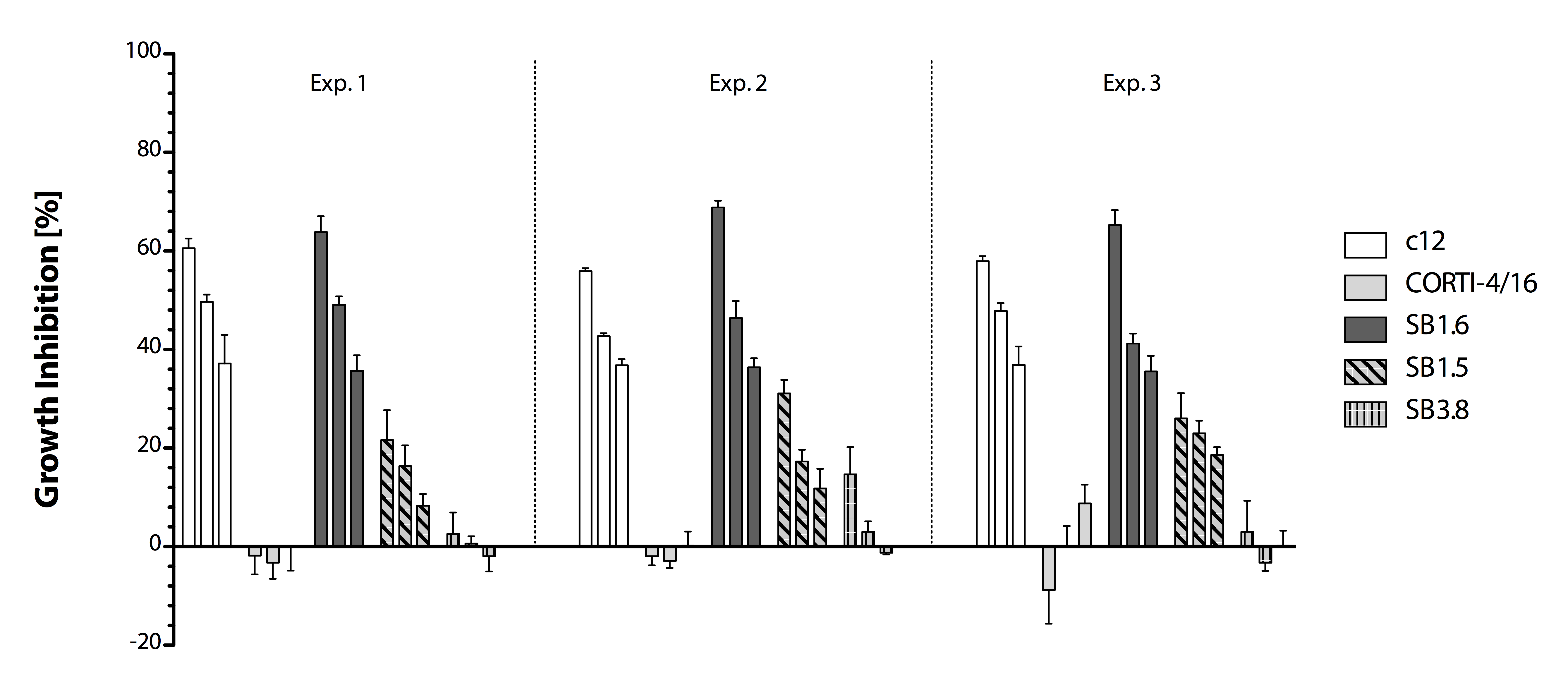

Supplement: Supplementary file 4 — 10.1186/s12936-016-1213-x In vitro parasite growth inhibitory activity of vaccine elicited anti-PfCyRPA mAbs was reproducible in independent experiments. Different batches of anti-PfCyRPA mAbs were produced and purified in the same way, and tested in independent in vitro growth inhibition assays. Reported are three independent experiments as representative examples of obtained results. Anti-PfCyRPA mAbs SB1.6 is shown as an example for an inhibitory mAb, mAb SB1.5 for a partially inhibitory mAb, and mAb SB3.8 for a non-inhibitory mAb. The anti-cortisol mAb CORTI-4/16 was used as negative control and the anti-PfCyRPA mAb c12 as positive control [32]. Each bar represents the mean of a triplicate experiment, and error bars indicate the SD. [file 12936_2016_1213_MOESM4_ESM.tiff]

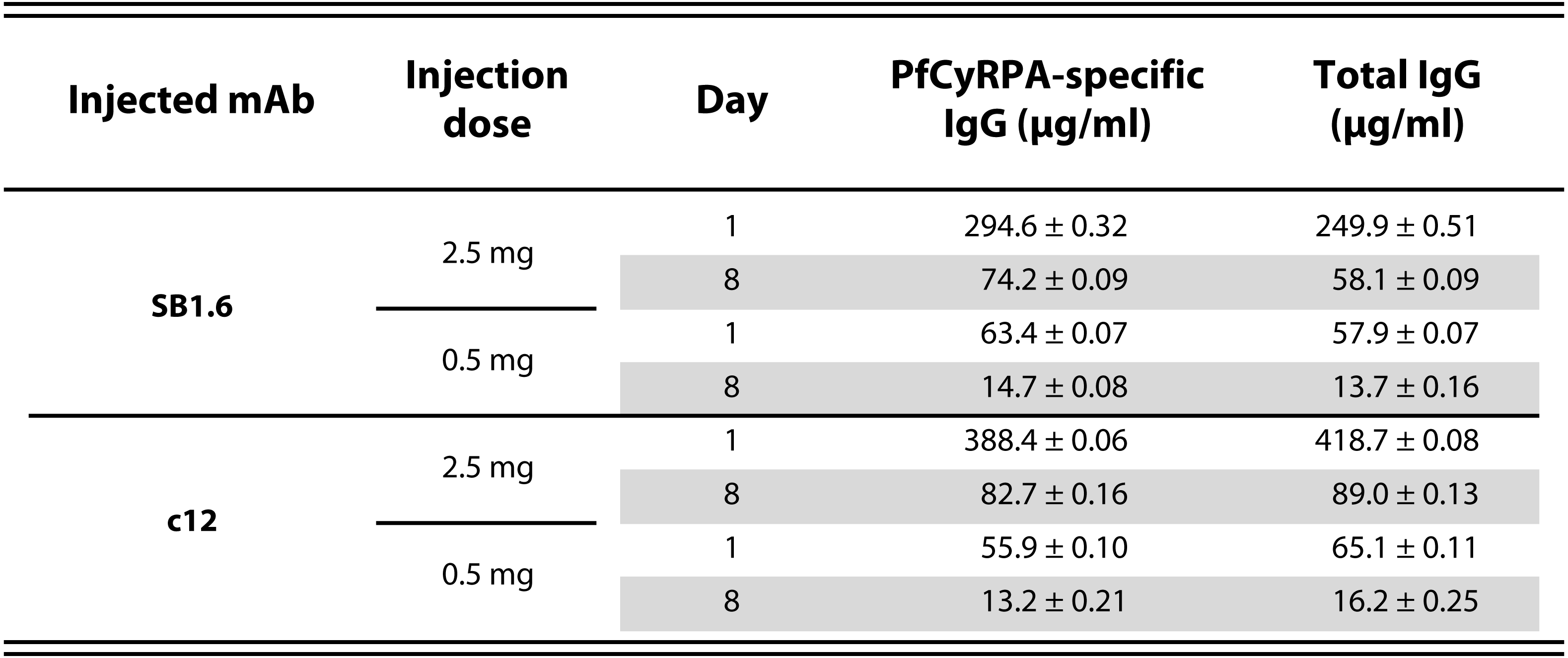

Supplement: Supplementary file 5 — 10.1186/s12936-016-1213-x Titration of administered mAbs in the serum of the passively immunized mice. The concentration of administered PfCyRPA-specific mAbs SB1.6 and c12 in the circulation was estimated by indirect ELISA on day one and eight after injection. For the detection of PfCyRPA-specific mAbs and total circulating IgGs, plates were coated with N-CyRPA or goat anti-mouse IgG (γ-chain specific) mAb (M1397, Sigma), respectively. After blocking, plates were incubated with dilutions of individual mouse serum. An HRP-conjugated goat anti-mouse IgG (γ-chain specific) Ab (A3673, Sigma) was used as secondary antibody and TMB as substrate. Standard curves were generated from known dilutions of SB1.6 and c12 mAbs and fit using a 4-PL logistic equation. Concentration of circulating mAbs was calculated by interpolating the absorbance values for the test sera from the standard curves. Reported values are means of three mice per group ± SD. [file 12936_2016_1213_MOESM5_ESM.tif]
